# Pharmacokinetics and pharmacodynamics of tiotropium solution and tiotropium powder in chronic obstructive pulmonary disease

**DOI:** 10.1002/jcph.215

**Published:** 2013-11-27

**Authors:** JM Hohlfeld, A Sharma, JA van Noord, PJG Cornelissen, E Derom, L Towse, V Peterkin, B Disse

**Affiliations:** 1 Fraunhofer Institute of Toxicology and Experimental Medicine (ITEM)Hannover, Germany; 2 Boehringer Ingelheim Pharma GmbH & Co. KGGermany, and affiliates in The Netherlands and UK; 3 Department of Respiratory Diseases, Atrium Medisch CentrumHeerlen, The Netherlands; 4 Department of Respiratory Diseases, Ghent University HospitalGhent, Belgium

**Keywords:** tiotropium Respimat® SMI, tiotropium HandiHaler®, COPD, pharmacokinetics, pharmacodynamics

## Abstract

The aim of the study was to characterize pharmacokinetics of tiotropium solution 5 µg compared to powder 18 µg and assess dose-dependency of tiotropium solution pharmacodynamics in comparison to placebo. In total 154 patients with chronic obstructive pulmonary disease (COPD) were included in this multicenter, randomized, double-blind within-solution (1.25, 2.5, 5 µg, and placebo), and open-label powder 18 µg, crossover study, including 4-week treatment periods. Primary end points were peak plasma concentration (C_max,ss_), and area under the plasma concentration–time profile (AUC_0–6h,ss_), both at steady state. The pharmacodynamic response was assessed by serial spirometry (forced expiratory volume in 1 second/forced vital capacity). Safety was evaluated as adverse events and by electrocardiogram/Holter. Tiotropium was rapidly absorbed with a median t_max,ss_ of 5–7 minutes postdosing for both devices. The gMean ratio of solution 5 µg over powder 18 µg was 81% (90% confidence interval, 73–89%) for C_max,ss_ and 76% (70–82%) for AUC_0–6h,ss_, indicating that bioequivalence was not established. Dose ordering for bronchodilation was observed. Powder 18 µg and solution 5 µg were most effective, providing comparable bronchodilation. All treatments were well tolerated with no apparent relation to dose or device. Comparable bronchodilator efficacy to powder18 µg at lower systemic exposure supports tiotropium solution 5 µg for maintenance treatment of COPD.

Inhaled long-acting bronchodilators are established options for the first-line pharmacologic management of chronic obstructive pulmonary disease (COPD).^1^,^2^ Tiotropium is the first once-daily, long-acting anticholinergic bronchodilator for the maintenance therapy of patients with COPD and is available in two formulations: an aqueous solution (5 µg) delivered via the Respimat® Soft Mist™ Inhaler (SMI) and a dry powder (18 µg) delivered via the breath-actuated HandiHaler® device (both formulations: Boehringer Ingelheim Pharma GmbH & Co KG, Ingelheim am Rhein, Germany). Numerous controlled trials, including either or both formulations, have shown that tiotropium provides at least a 24-hour sustained improvement in airflow, reduced hyperinflation, and shortness of breath, as well as a reduced risk of exacerbations and associated hospitalizations, resulting in an increased health-related quality of life.^3^–^10^ The multidose Respimat® SMI provides a high level of satisfaction in COPD patients due to its inhalation and handling characteristics.^11^

The clinical development program of tiotropium solution 5 µg included two 4-week, placebo-controlled, crossover studies, which were primarily designed to compare tiotropium solution and powder in terms of lung function improvement over a 12-hour observation period.^10^ The prespecified pooled analysis of these two studies revealed that tiotropium solution 5 and 10 µg were noninferior to tiotropium powder 18 µg in terms of the primary efficacy end point, trough forced expiratory volume in 1 second (FEV_1_) assessed at the end of the 24-hour dosing interval, and a series of secondary efficacy end points (forced vital capacity [FVC], peak expiratory flow, rescue medication use). All tiotropium treatments were well tolerated. In a subset of the study population, urine and limited blood samples were collected for pharmacokinetic (PK) assessments.

Both studies incorporated a double-dummy design, meaning that all patients inhaled from both inhalers prior to the spirometric assessments and sampling for PK.^10^ While this design is preferred in a therapeutic trial focusing on efficacy and safety, it has disadvantages for PK assessments as early blood sampling is compromised by the additional time required and timing imprecision for patients to properly inhale from two devices. Therefore, the present PK-focused study was undertaken to complement the existing comparative PK data in which blood sampling was limited to predose, 10 minutes, and 1 and 6 hours after tiotropium dosing. The first postinhalation sample at 10 minutes provided the highest mean plasma level, which may not accurately represent the maximum plasma concentration at steady state (C_max,ss_) because earlier time points with potentially higher values were not assessed. The open-label HandiHaler® and double-blind within Respimat® design in the current study allowed one inhaler precisely timed serial blood sampling, starting as early as 2 minutes following inhalation, and allowed reliable estimation of time to maximum plasma concentration and C_max,ss_.

The primary objective of this open-label, randomized, double-blind within solution (tiotropium 1.25, 2.5, 5 µg and placebo), 5-way crossover study including 4-week treatment periods, was to characterize the PK of tiotropium solution 5 µg in comparison to tiotropium powder 18 µg. Secondary objectives were to characterize the PK of tiotropium solution 1.25 and 2.5 µg, additional PK end points and to assess the dose dependency of pharmacodynamics of tiotropium solution (spirometry) compared to placebo.

## Methods

### Study Design

This study (#205.458 [sponsor's protocol number], clinicaltrials.gov: NCT01222533) was designed as a multicenter, placebo-controlled, randomized, double-blind (within Respimat® SMI), 5-way, crossover trial with 4-week treatment periods of placebo and once-daily tiotropium doses of 1.25, 2.5, and 5 µg (two puffs of 0.625, 1.25, and 2.5 µg, respectively) as aqueous solution for inhalation via the Respimat® SMI and open-label tiotropium 18 µg as dry powder for inhalation via HandiHaler®. All doses were administered in the morning between 7:00 and 10:00 am. There was no washout period between the 4-week treatment periods, but 4 weeks' treatment is sufficient to reach PK and pharmacodynamic (PD) steady state before the test days.^12^,^13^ Compliance with medication use was recorded.

Following an initial screening visit to assess eligibility and a 1-week run-in period, eligible patients were assigned to a treatment sequence for evaluation of PK after 26 days and PD after 28 days at the end of each of the five treatment periods. Medications for the routine management of COPD were permitted throughout the study period, provided that dosages had been stabilized for at least 6 weeks prior to screening. These included oral corticosteroids at low doses (≤10 mg prednisolone daily or equivalent), inhaled corticosteroids, mucolytic agents (not containing bronchodilators), antihistamines, antileukotrienes (unless prescribed for asthma or excluded allergic conditions), and twice-daily inhaled long-acting β_2_-agonists (LABAs), such as formoterol or salmeterol. LABAs were withdrawn at least 24 hours prior to each PK test day and at least 48 hours prior to pulmonary function test (PFT) days. Open-label salbutamol was provided and patients were instructed to use it as needed for acute symptom relief and to withdraw its use at least 8 hours prior to any clinic test day. Short-acting anticholinergics were permitted during the screening and run-in periods, and withdrawn at least 8 hours prior to PFTs. Patients discontinued once-daily LABA (indacaterol), long-acting anticholinergics, oral β_2_-adrenergic agonists, and β-blockers at least 2 weeks prior to the screening visit in order to have at least a 3-week washout period prior to randomization. Medications to control acute exacerbations were allowed during randomized treatment according to medical need.

Prior to the start of the study, the clinical trial protocol and other relevant study documentation were reviewed by the Independent Ethics Committee of the participating centers (University Hospital Gent [central committee for Belgian sites]; De Videnskabsetiske Komitéer for Region Hovedstaden, Hillerød, Denmark [central committee for Danish sites]; HUS Medisiininen Eettinen toimikunta, Helsinki, Finland; Ethikkommission der Medizinischen Hochschule, Hannover, Germany; METC Atrium-Orbis-Zuyd, Heerlen, the Netherlands [central committee for Dutch sites]). The study was conducted in accordance with the Declaration of Helsinki (1996). Written informed consent was obtained prior to any study-related procedure.

### Patients

Patients eligible for inclusion were men or women aged ≥40 years, current, or ex-smokers with a smoking history of ≥10 pack-years, and diagnosed with COPD^2^ (postbronchodilator FEV_1_ <80% of predicted^14^ and postbronchodilator FEV_1_/FVC ratio <70%). The main exclusion criteria included significant diseases other than COPD, diagnosis of asthma, elevated blood eosinophil count (≥600 mm^3^), recent history (i.e., 6 months or less) of myocardial infarction, unstable or life-threatening cardiac arrhythmia requiring intervention or change in drug therapy during the past year, hospitalization for cardiac failure during the past year, malignancy requiring resection, radiation therapy, or chemotherapy within the last 5 years. Patients with a respiratory infection or COPD exacerbation in the 6 weeks prior to screening or during the run-in period were allowed to be randomized 6 weeks following recovery from the event.

### Study Assessments

On day 26 of each treatment period, blood samples were collected 5 minutes before dosing and during a 6-hour period at 2, 5, 7, 9, 12, 15, 20, 30, 40, 60 minutes, and 2, 4, and 6 hours post dosing. In addition, urine for the assessment of tiotropium excretion was collected −1 to 0 hours (predosing), and 0–2 and 2–6 hours postdosing. Tiotropium concentrations were determined by a validated assay using high-performance liquid chromatography coupled to tandem mass spectrometry (Nuvisan GmbH, Neu-Ulm, Germany). Compared to the assay previously used,^8^ the refined assay had a lower limit of quantification of 1.0 pg/mL.

PFTs were conducted according to the American Thoracic Society criteria.^15^ At screening, prebronchodilator and qualifying postbronchodilator spirometric tests (FEV_1_, FVC) were performed (45 minutes after inhalation of four puffs of salbutamol 100 µg metered-dose inhaler). After completion of the run-in period, the study baseline FEV_1_ and FVC were determined before inhalation of the first dose of study medication. On day 28 of each treatment period, 2 days after the PK test day, serial 6-hour spirometry was conducted at 10 minutes before and at 30 and 60 minutes, and 2, 3, 4, 5, and 6 hours after inhalation of study medication. Safety evaluations included adverse event (AE) recording throughout the study and ECG/Holter monitoring on the 6-hour PFT days.

### Statistical Analyses

The primary end points for PK evaluation of tiotropium solution 5 µg versus tiotropium powder 18 µg at PK/PD steady state were C_max,ss_ and the area under the plasma concentration–time curve profile from 0 to 6 hours at steady state (AUC_0–6h,ss_). These primary PK end points were expected to follow a log-normal distribution, whereas the FEV_1_- and FVC-based secondary efficacy end points (AUC_0–6h_, trough and AUC_0–3h_) were expected to follow a normal distribution. The average FEV_1_ and FVC (AUC_0–3h_, AUC_0–6h_) were calculated as the AUC from 0 to 3 or 6 hours, respectively, using the trapezoidal rule divided by the corresponding duration (i.e., 3 or 6 hours) to give the result in liters. The predose or trough FEV_1_ (or FVC) value was assigned to zero time and defined as the measurement before administration of the last once-daily dose of study drug at the end of each 4-week treatment period. Based upon design and distributional considerations, analysis of variance (ANOVA) suitable for crossover trials was employed, using a mixed-effects model. The ANOVA model accounted for the following sources of variation: “sequence,” “patients within sequences,” “period,” and “treatment.” “Patients within sequences” was considered random, while the other effects were fixed.

C_max,ss_ and AUC_0–6h,ss_ were log transformed prior to fitting the ANOVA model. The difference between the expected means for log(T)–log(R) (i.e., test treatment tiotropium solution 5 µg, reference treatment tiotropium powder 18 µg) was estimated by the difference in the corresponding least square means (point estimate) and 2-sided 90% confidence intervals (CIs) were computed. These quantities were back transformed to the original scale to give the geometric mean (gMean) and interval estimates for the median intrapatient ratio between the response under test (tiotropium solution 5 µg) and response under reference (tiotropium powder 18 µg). Bioequivalence of the two inhaled products was assumed if the 90% CIs for each of the ratios of the gMeans of C_max,ss_ and AUC_0–6h,ss_ of the test (tiotropium solution 5 µg) and reference product (tiotropium powder 18 µg) were contained in the predefined acceptance interval of 80–125%.^16^

The spirometric efficacy of tiotropium solution 2.5 or 5 µg was established by a 2-sided test at α = 0.05. A 1-sided test at α = 0.025 was performed to see if tiotropium solution 1.25 µg was an ineffective dose compared to placebo. In order for tiotropium solution 1.25 µg to be declared an ineffective dose compared to placebo, the 95% CI for the treatment difference had to lie entirely below the prespecified limit of 0.1 L.

The PK end point analyses were based on the PK set, while analyses of the efficacy (spirometry) end points were performed for the full analysis set (FAS) (i.e., patients who had received at least one dose of study medication and for whom ontreatment data for at least one efficacy end point was available) and the per-protocol set (FAS and did not experience a relevant important protocol violation). Evaluation of safety end points (AEs, including patients who received at least one dose of study medication, and ECG/Holter) were summarized descriptively for the treated set.

In order to determine whether tiotropium solution 1.25 µg was an ineffective dose compared to placebo, 120 completed patients were required, assuming a type I error rate (α) of 0.025 (1-sided), 90% power, a standard deviation of the within-patient treatment differences of 0.167 L,^3^,^8^,^10^ and a treatment difference of 0.1 L compared to placebo. After accounting for a dropout rate of 15%, 142 patients were planned to be randomized.

## Results

**Figure 1:**
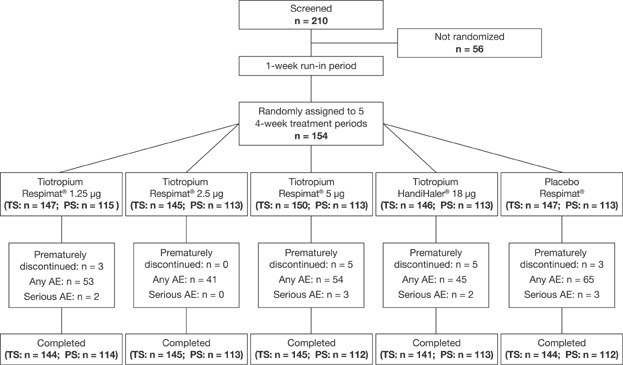
Flow diagram, design, and patient disposition. A total of 210 patients were initially enrolled and screened. Following a 1-week run-in period, 154 patients were randomized to five treatment groups, for a 4-week period. The number of patients in each treatment group that comprised the treatment set (TS) and the pharmacokinetic set (PS) at randomization and study end is indicated. The number of patients in each treatment group who prematurely discontinued treatment, reported any adverse event (AE) or serious AE is also indicated in the figure. The result showed that tiotropium delivered either as a powder or solution was well tolerated.

A total of 154 patients were randomized to treatment. Table1 provides the demographics and baseline characteristics at screening for the patients included in the efficacy and safety analyses. The majority of patients were male (76%), current smokers (64%) with a mean prebronchodilator FEV_1_ of 1.44 L (48% of predicted normal), and a mean COPD duration of 9 years; comparable data were seen for the subgroup included in the PK assessments. Patient disposition and the number of patients randomized to each 4-week treatment group are provided in Figure 1; 140 patients (91%) completed the study.

**Table 1:** Demographics and Characteristics at Screening and Randomization Visits

Variables^a^	Treated set (N = 154)
Screening visit	
Male (%)	76.0
Age (years)	63.1 (7.8)
Smoking status:	
Ex-smoker (%)	36.4
Current smoker (%)	63.6
Smoking history (pack-years)	47.5 (21.5)
FEV_1_ prebronchodilator (L)	1.4 (0.5)
Percent predicted normal	48.1 (14.3)
FVC prebronchodilator (L)	3.3 (1.0)
FEV_1_/FVC prebronchodilator (%)	44.5 (10.9)
FEV_1_ postbronchodilator (L)	1.6 (0.6)
Percent predicted normal	54.4 (14.5)
FVC postbronchodilator (L)	3.6 (1.0)
FEV_1_/FVC postbronchodilator (%)	45.6 (11.0)
Reversibility FEV_1_ (L)	0.2 (0.1)
Reversibility FEV_1_ (%)	14.7 (10.9)
COPD severity according to GOLD (%)	
Moderate (50 to <80%)	63.0
Severe (30 to <50%)	31.2
Very severe (<30%)	5.8
Prestudy medication for COPD, n (%)	
Inhaled short-acting anticholinergics	10 (6.5)
Inhaled long-acting anticholinergics	79 (51.3)
Inhaled short-acting β_2_-adrenergics	59 (38.3)
Inhaled long-acting β_2_-adrenergics	90 (58.4)
Inhaled corticosteroids	81 (52.6)
Oral corticosteroids	2 (1.3)
Theophylline	3 (1.9)
Randomization visit (study baseline)	
FEV_1_ (L)	1.4 (0.6)
FEV_1_ (% predicted normal)	47.4 (14.4)
COPD medication during randomized study period, n (%)	
Inhaled long-acting β_2_-adrenergics	90 (58.4)
Inhaled corticosteroids	80 (51.9)
Oral corticosteroids	1 (0.6)

COPD, chronic obstructive pulmonary disease; FEV_1_, forced expiratory volume in 1 second; FVC, forced vital capacity; GOLD, Global Initiative for Chronic Obstructive Lung Disease.

Medications permitted throughout the entire study period included oral corticosteroids at minimal doses (≤20 mg), orally inhaled corticosteroids, mucolytic agents (not containing bronchodilators), antihistamines, antileukotrienes, and leukotriene receptor antagonists (unless prescribed for asthma or excluded allergic conditions) and orally inhaled twice daily long-acting β2-adrenergic agents (formoterol or salmeterol).

a Data are mean (±SD) unless otherwise stated.

### Pharmacokinetics

**Figure 2:**
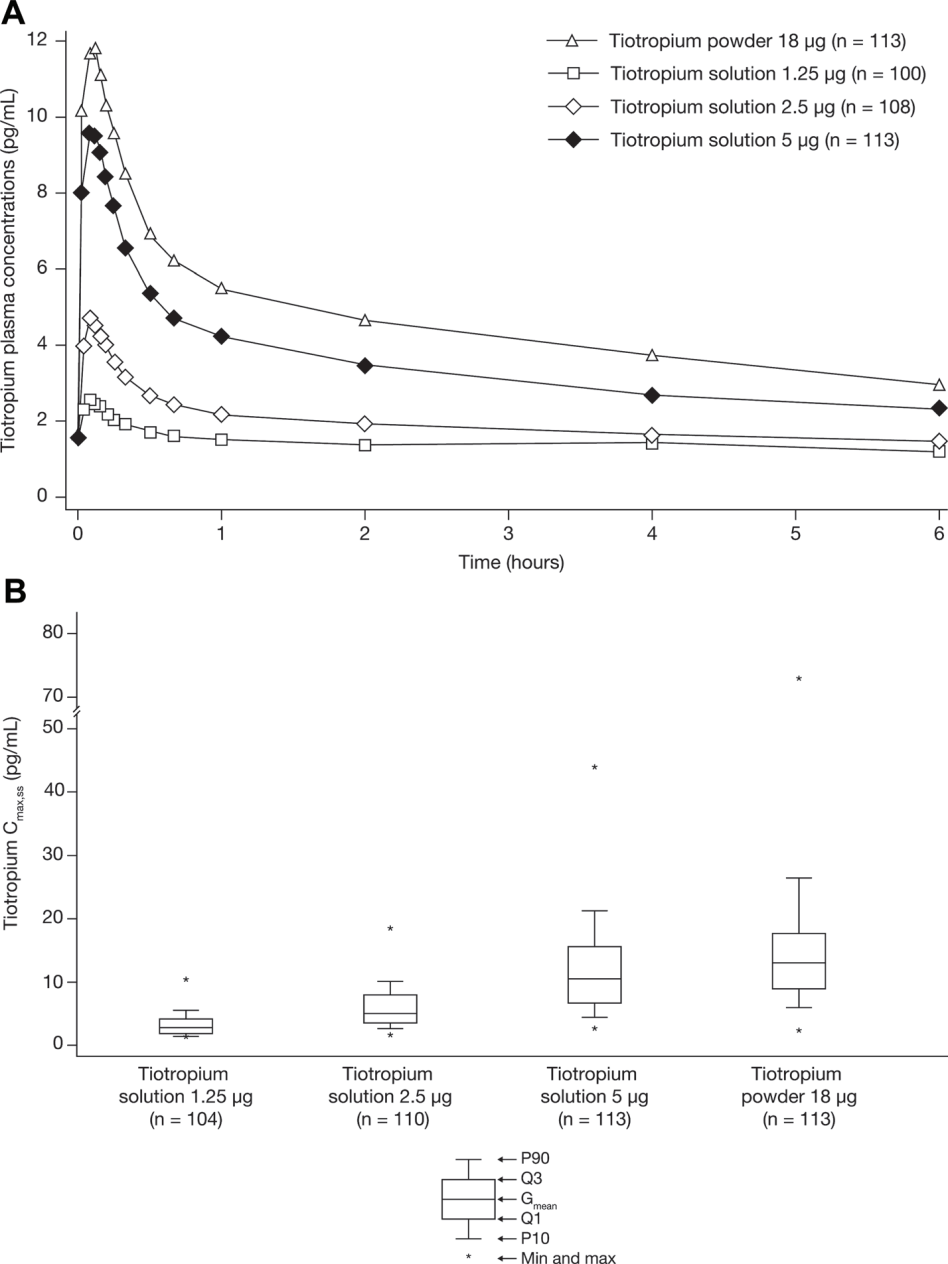
Clinical study. (A) Geometric mean tiotropium plasma concentration–time profile following multiple inhalations as solution or as powder. (B) Comparison of maximum tiotropium plasma concentrations at steady state (C_max,ss_) by dose and device (solution versus powder). On day 26 of each treatment period, blood samples were collected 5 minutes before dosing and during a 6-hour period at 2, 5, 7, 9, 12, 15, 20, 30, 40, 60 minutes, and 2, 4, and 6 hours postdosing. Tiotropium concentrations were determined by a validated assay using high-performance liquid chromatography coupled to tandem mass spectrometry. (A) Mean plasma concentrations were (slightly) lower with tiotropium solution 5 µg compared with tiotropium powder 18 µg. (B) There was a dose-dependent increase in tiotropium plasma exposure as determined by C_max_.

The gMean plasma concentration–time profiles following 26 days of once-daily dosing with the three tiotropium solution doses (1.25, 2.5, or 5 µg) or tiotropium powder 18 µg are shown in Figure 2A. Following inhalation with tiotropium solution, there was a dose-dependent increase in tiotropium plasma exposure as determined by C_max,ss_ (Figure 2B), and the amount of drug eliminated in Ae_0–6,ss_ and AUC_0–1,ss_ values (Table2). The PK parameters obtained with both tiotropium formulations are summarized by treatment group in Table2.

**Table 2:** PK Parameters by Treatment Group

Variable	Tiotropium solution									Tiotropium powder		
	1.25 µg			2.5 µg			5.0 µg			18.0 µg		
	n	gMean	gCV (%)	n	gMean	gCV (%)	n	gMean	gCV (%)	n	gMean	gCV (%)
C_max,ss_ (pg/mL)	104	2.8	53.0	110	5.1	61.8	113	10.5	66.4	113	12.9	64.6
C_max,ss,norm_ (pg/mL/µg)	104	2.3	53.0	110	2.0	61.8	113	2.1	66.4	113	0.7	64.6
AUC_0–6h,ss_ (pg · h/mL)	22	10.0	25.3	76	12.8	29.9	107	22.1	47.8	113	28.4	52.4
AUC_0–6h,ss,norm_ (pg · h/mL/µg)	22	8.0	25.3	76	5.1	29.9	107	4.4	47.8	113	1.6	52.4
AUC_0–1h,ss_ (pg · h/mL)	61	2.1	32.3	97	3.2	44.4	112	6.1	58.3	113	7.8	54.9
AUC_0–1h,ss,norm_ (pg · h/mL/µg)	61	1.7	32.3	97	1.3	44.4	112	1.2	58.3	113	0.4	54.9
Ae_0–6h,ss_ (ng)	108	88.7	68.0	110	177	68.0	107	387	65.9	109	522	53.8
f_0–6h,ss_ (%)	108	7.09	68.0	110	7.1	68.0	107	7.8	65.9	109	2.9	53.8
t_max,ss_ (h)^a^	104	0.100	0.0330–2.00	110	0.0830	0.0330–6.00	113	0.117	0.0330–0.333	113	0.117	0.0330–1.00

Ae_0–6h,ss_, amount of drug excreted in urine from 0 to 6 hours at steady state; AUC_0–1h,ss_, area under the plasma concentration–time profile from 0 to 1 hour at steady state; AUC_0–1h,ss,norm_, normalized area under the plasma concentration–time profile from 0 to 6 hours at steady state; AUC_0–6h,ss_, area under the plasma concentration–time profile from 0 to 6 hours at steady state; AUC_0–6h,ss,norm_, normalized area under the plasma concentration–time profile from 0 to 6 hours at steady state; C_max,ss_, peak plasma concentration at steady state; C_max,ss,norm_, normalized peak plasma concentration at steady state; f_0–6h,ss_, fraction of the delivered dose excreted in urine from 0 to 6 hours at steady state; gCV, geometric coefficient of variation; gMean, geometric mean; t_max,ss_, time to C_max_ at steady state.

a Median and range.

Following inhalation from either device, tiotropium was rapidly absorbed with similar t_max,ss_ values for all treatments ranging between 5 and 7 minutes postdosing. The C_max,ss_ estimation is reliable because quantifiable tiotropium plasma levels were obtained from at least at one time point before t_max,ss_ was reached. From C_max,ss_, plasma concentrations declined rapidly for 30 minutes followed by a more gradual decline until 6 hours postdosing. The shape of the tiotropium plasma profile was similar following inhalation from both devices. Mean plasma concentrations were (slightly) lower with tiotropium solution 5 µg compared with tiotropium powder 18 µg (Figure 2A).

### Bioequivalence Testing of Solution 5 µg Versus Powder 18 µg

Bioequivalence was not observed based on a comparison of the C_max,ss_ and AUC_0–6,ss_ values. The corresponding gMean ratio for the C_max,ss_ values of 10.5 pg/mL (5 µg) and 12.9 pg/mL (18 µg) was 81% and the 90% CI (73–89%) was below the prespecified acceptance interval of 80–125%. The gMean AUC_0–6h,ss_ values were 22.1 pg · h/mL following tiotropium solution 5 µg and 28.4 pg · h/mL following tiotropium powder 18 µg. The corresponding ratio of the AUC_0–6h,ss_ values was 76% (90% CI: 70–82%) and again, the 90% CI was below the prespecified acceptance interval of 80–125%, indicating that bioequivalence was not established. The difference of 6.3 pg · h/mL (22%) found between both drug products in terms of AUC_0–6h,ss_ was supported by Ae_0–6h,ss_. In the time interval 0–6 hours postdose, the amount of renally excreted tiotropium was 26% lower following the administration of tiotropium solution 5 µg compared with tiotropium powder 18 µg (Table2).

### Evaluation of Dose Proportionality (Solution Doses 1.25, 2.5, or 5 µg)

The C_max,ss_ and the Ae_0–6h,ss_ values increased in proportion to the inhaled tiotropium solution dose. Based on the plasma levels reliably measured until 1 hour postinhalation (AUC_0–1h,ss_), exposure to tiotropium increased in a dose–proportional manner in line with the C_max,ss_ and Ae_0–6,ss_ values.

### Spirometry

**Figure 3:**
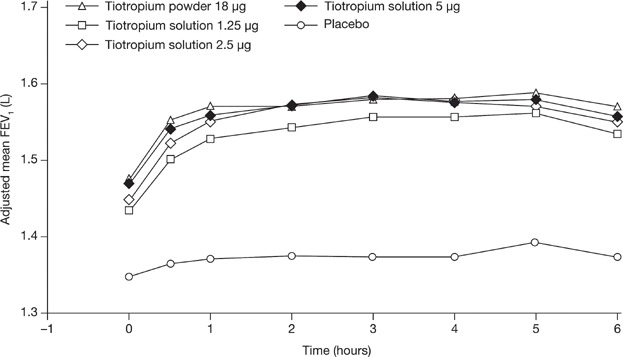
Adjusted mean FEV_1_ over 6 hours at day 28. On day 28 of each treatment period, 2 days after the pharmacokinetic (PK) test day, serial 6-hour spirometry was conducted at 10 minutes before and at 30 and 60 minutes, and 2, 3, 4, 5, and 6 hours after inhalation of study medication. Tiotropium solution doses 1.25, 2.5, and 5 µg and tiotropium powder 18 µg provided significantly (*P* < .0001) greater bronchodilation compared to placebo in all spirometric end points. Within the first hour postdosing, tiotropium solution 2.5 µg provided less bronchodilatory effects compared with both solution 5 µg and powder 18 µg, while solution 5 µg and powder 18 µg were similar. FEV_1_, forced expiratory volume in 1 second.

The FEV_1_ time–response curves after inhalation of three doses (1.25, 2.5, and 5 µg) of tiotropium solution and tiotropium powder 18 µg are shown in Figure 3; end points derived from the 6-hour profiles are presented in Table3.

**Table 3:** Clinical Study: FEV_1_ Results by Treatment Group

	Treatment group				
	Placebo (n = 143)	Solution 1.25 µg (n = 143)	Solution 2.5 µg (n = 144)	Solution 5 µg (n = 143)	Powder 18 µg^b^ (n = 142)
Day 29 FEV_1_ AUC_0–6h_ (mL)					
Adjusted mean (SE)	1,371 (46)	1,535 (46)	1,556 (46)	1,562 (46)	1,567 (46)
Comparison versus placebo^a^					
Adjusted mean (SE)		165 (12)	185 (12)	191 (12)	196 (12)
95% CI		141, 189	161, 209	167, 216	172, 220
*P*-value		<.0001^a^	<.0001	<.0001	<.0001
Comparison versus powder 18 µg					
Adjusted mean (SE)		−31 (12)	−11 (12)	−5 (12)	
95% CI		−55, −7	−35, 13	−29, 19	
Trough FEV_1_ (mL)					
Adjusted mean (SE)	1,345 (45)	1,432 (45)	1,446 (45)	1,466 (45)	1,473 (45)
Comparison versus placebo^a^					
Adjusted mean (SE)		87 (14)	101 (14)	121 (14)	128 (14)
95% CI		60, 114	74, 128	94, 148	101, 155
*P*-value		<.0001^a^	<.0001	<.0001	<.0001
Comparison versus powder 18 µg					
Adjusted mean (SE)		−41 (14)	−27 (14)	−7 (14)	
95% CI		−68, −14	−55, 0	−34, 20	
Day 29 FEV_1_ AUC_0–3h_ (mL)					
Adjusted mean (SE)	1,366 (46)	1,521 (46)	1,546 (46)	1,553 (46)	1,558 (46)
Comparison versus placebo^a^					
Adjusted mean (SE)		155 (13)	180 (13)	187 (13)	191 (13)
95% CI		130, 180	155, 205	162, 211	167, 216
*P*-value		<.0001^a^	<.0001	<.0001	<.0001
Comparison versus powder 18 µg					
Adjusted mean (SE)		−36 (13)	−11 (13)	−5 (13)	
95% CI		−61, −12	−36, 13	−29, 20	

AUC_0–3h_, area under the curve from 0 to 3 hours; AUC_0–6h_, area under the curve from 0 to 6 hours; CI, confidence interval; FEV_1_, forced expiratory volume in 1 second; SE, standard error.

a Superiority tests for tiotropium solution 1.25 µg versus placebo were not predefined; in order for tiotropium solution 1.25 µg to be declared an ineffective dose compared to placebo, the 95% CI for the treatment difference had to lie entirely below the prespecified limit of 0.1 L.

b Tiotropium powder 18 µg was not blinded.

Tiotropium solution doses 1.25, 2.5, and 5 µg and tiotropium powder 18 µg provided significantly (*P* < .0001) greater bronchodilation compared to placebo in all spirometric end points. This hypothesis was only predefined for the doses 2.5 and 5 µg. It was hypothesized that the 1.25-µg dose would be no more effective than placebo. This would have been assumed if the upper limit of the 95% CI for the difference (1.25 µg to placebo) was below the predefined limit of 0.1 L, which was not the case. However, effects with the lowest dose of tiotropium solution (1.25 µg) were statistically inferior to the 18-µg dose of tiotropium powder in all FEV_1_-based end points. Considering FEV_1_ AUC_0–6h_ and FEV_1_ AUC_0–3h_, the 2.5- and 5-µg dose of tiotropium solution were the most effective and comparable with tiotropium powder 18 µg. However, within the first hour postdosing, tiotropium solution 2.5 µg provided fewer bronchodilatory effects compared with both tiotropium solution 5 µg and tiotropium powder 18 µg, while tiotropium solution 5 µg and tiotropium powder 18 µg were similar. In addition, we observed a statistically significant higher trough FEV_1_ following treatment with tiotropium powder 18 µg compared with tiotropium solution 2.5 µg, but not with tiotropium powder 18 µg compared with tiotropium solution 5 µg (Table3).

Improvements observed in the 6-hour FVC time-response curves mirror the FEV_1_ findings. Irrespective of the inhaler, significantly higher responses in FVC were observed with all four tiotropium treatments compared to placebo_,_ with the most pronounced and similar bronchodilatory effects to tiotropium powder 18 µg seen following the 2.5- and 5-µg doses of tiotropium solution. Again, a numerically lower response in FVC was found following the 2.5-µg dose at the end of the 24-hour dosing interval (trough) and within the first hour postdosing.

Individual trough FEV_1_ and FEV_1_ AUC_0–3h_ values were compared with the corresponding trough tiotropium plasma concentrations (C_pre,ss_) and the maximum plasma concentrations at steady state (C_max,ss_), respectively (Supplementary Figures 1 and 2). No correlation was found between the response in FEV_1_ and the tiotropium plasma concentration.

### Safety

Six patients (3.9%) prematurely discontinued study medication due to an AE, five of whom subsequently withdrew from the study and one continued with the next two treatment periods and completed the study. AEs leading to study discontinuation occurred in two patients on placebo (worsening of COPD, development of hernia) and one patient on tiotropium solution 5 µg (worsening of COPD), one patient on tiotropium solution 1.25 µg (worsening of baseline condition esophageal reflux), and two patients on tiotropium powder 18 µg (worsening of COPD and pneumonia). The frequency of AEs was balanced between the tiotropium treatment periods and similar to the placebo period with the exception of complaints of dyspnea and worsening of COPD, which were more frequently reported during the placebo period. In total, 10 patients experienced a serious AE. Of these, three in the placebo period (one with a hemorrhoidal hemorrhage, two with an exacerbation of COPD), three while on tiotropium solution 5 µg (colitis ischemic, pancreatitis, renal tubular necrosis/azotemia), two on tiotropium solution 1.25 µg (cerebrovascular accident/spinal column stenosis, prostate cancer), and two on tiotropium powder 18 µg (exacerbation of COPD, pneumonia). None of these events was considered related to study medication. There was no apparent treatment-related effect or pattern in the ECG/Holter assessments, which will be separately reported in the context of a broader analysis including several studies.

## Discussion

Tiotropium powder 18 µg and tiotropium solution 5 µg are different formulations for the same active ingredient. While developed and profiled in standalone, full programs, the delivered dose of tiotropium solution was adjusted because of the high device efficiency to a value below the nominal dose of tiotropium powder to achieve comparability in efficacy and systemic exposure. For inhaled medications, systemic availability and exposure is typically discussed in the context of safety, whereas it is not considered predictive for efficacy. Many inhaled medications, among them tiotropium,^17^ are absorbed rapidly from the lungs, so that assessment of t_max_ and C_max_ is critical and requires precise and early sampling. Earlier efficacy and PK studies^8^,^18^ had shown numerically higher or similar exposure to tiotropium following treatment with tiotropium solution 5 µg compared with tiotropium powder 18 µg. Taking up a numerical imbalance in mortality of tiotropium solution compared to placebo,^19^ recent systematic reviews and meta-analyses of randomized trials, including 1-year studies,^3^,^4^,^8^,^9^ have reported a potential association between the use of tiotropium solution 5 µg and an increased risk of mortality in COPD patients.^20^–^22^ Higher peak and overall tiotropium exposure following inhalation from the Respimat® SMI has been suggested to be causally related to this observation.^22^ For this reason, optimized PK and efficacy comparison of the 2 tiotropium formulations is very important. The crossover and open-label design (instead of double-dummy) for comparison across formulations is an essential feature of this study allowing precise timing as early as 2 minutes following inhalation. The study included an elderly COPD patient population and allowed typical co-medication. Since both tiotropium formulations are indicated for the maintenance therapy in COPD, the bioequivalence primary end points were determined at PK steady state, the most relevant time point. After chronic once-daily inhalation of tiotropium powder by COPD patients, PK steady state is reached after 2–3 weeks with no accumulation thereafter.^12^ From this perspective, the study is representative for clinical use and defines a high standard for investigating inhaled medications.

The key result of this extensive comparative PK study in patients with COPD demonstrated a lower systemic exposure and availability of tiotropium following treatment with tiotropium solution 5 µg compared with tiotropium powder 18 µg (gMean for C_max,ss_ 19% lower and for AUC_0–6h,ss_ 24% lower). Although we observed widely overlapping ranges of individual plasma levels, formal testing revealed that bioequivalence was not established. In line with the ratio of 76% for solution versus powder in terms of plasma AUC_0–6h,ss_, the amount of renally excreted tiotropium over the 6-hour postdosing period was 26% lower for tiotropium solution 5 µg compared with tiotropium powder 18 µg. Thus, the renal excretion based on a 6-hour postdose urine collection period appeared to be a reliable indicator of plasma AUC_0–6h,ss_ values.

The dose-normalized total exposure based on AUC_0–6h,ss_ for the 1.25-µg dose was higher than that of other dose groups. However, this value may not be representative because tiotropium plasma concentrations were below the limit of quantification by 1 hour postdose for a majority of patients and hence were not included in estimating the average. Systemic exposure to tiotropium solution 1.25–5 µg increased in a dose–proportional manner based on C_max,ss_, Ae_0–6h,ss_, and AUC_0–1h,ss_ values.

From these results, it is evident that the chosen design was successful in capturing the actual peak plasma concentration as quantifiable tiotropium plasma levels were obtained at least one time point before reaching t_max,ss_. Due to the early and serial sampling postdosing, a reliable estimate of peak exposure (C_max,ss_) was achieved in this large COPD population. Furthermore, the complete plasma concentration–time profile was adequately described during the 6-hour observation period; the refined assay with a low limit of quantification (1.0 pg/mL) allowed quantifying of tiotropium in plasma in the majority of patients up to 6 hours postdosing with solution 2.5–5 µg and powder 18 µg.

As already mentioned, previously conducted PK studies had shown similar^18^ or numerically higher^8^ exposure with tiotropium solution 5 µg compared with tiotropium powder 18 µg. When discussing the differences in results, it is important to take aspects of the study design into account. In the present study, the focus on PK allowed precise timing, early and serial blood sampling over a 6-hour period, while the previously conducted studies that focused on bronchodilator efficacy had only 10-minute sampling and less precise timing with solution inhaled after powder. The published result of the prespecified pooled analysis of two studies^8^ reported a numerically higher gMean value of the plasma concentration at 10 minutes postdosing for tiotropium solution 5 µg compared with tiotropium powder 18 µg (11.5 and 8.49 pg/mL, respectively). The closest comparison using the current dataset is by selecting the gMean 9-minute postdose values, which showed a numerically lower gMean value for tiotropium solution 5 µg compared with tiotropium powder 18 µg (9.14 vs. 11.2 pg/mL, respectively). The difference between study sets may be due to differences in the study populations or study design differences, although an overall difference of 2–3 pg/mL in gMean tiotropium plasma levels following inhalation can be considered small. Evaluation of the intra-individual comparison between devices/formulations did not suggest a relevant difference in bioavailability. Another PK investigation in Japanese COPD patients showed comparable systemic exposure with the two tiotropium formulations.^18^

The apparent small difference in systemic exposure based on plasma and urine levels between tiotropium solution 5 µg and tiotropium powder 18 µg in our study is not reflected in the bronchodilator efficacy. Comparable spirometric improvements were observed for both approved tiotropium formulations. The study was not designed and powered for comparisons between each tiotropium solution dose (1.25–5 µg) and tiotropium powder 18 µg. Tiotropium solution 5 µg was shown to be the most comparable dose to tiotropium powder 18 µg in all FEV_1_ and FVC-based end points. Tiotropium solution 2.5 µg showed comparable bronchodilator efficacy in FEV_1_ and FVC to tiotropium powder 18 µg from 1 to 6 hours postdosing, while improvements were lower than the comparator in the first 60 minutes postinhalation. The mean FEV_1_ at the end of the 24-hour dosing interval (trough value) for tiotropium solution 2.5 µg was again significantly lower than the comparator (trough FVC numerically lower). Previously reported studies^3^,^8^ have shown non-significant differences between 5 and 10 µg in terms of pulmonary effects, which may indicate that tiotropium solution 5 µg is at the dose–response plateau.

All active treatments were well tolerated, similar to placebo and consistent with previous trials with similar treatment periods.^8^,^18^ There were no new safety signals and no fatal events. The current extensive study confirms in a large COPD population the results from the earlier comparative PK studies and does not indicate any relevant difference in absorption rate and extent of exposure between tiotropium solution 5 µg and tiotropium powder 18 µg. Therefore, the suggested causal relation of a mortality imbalance to differential tiotropium exposure is unsubstantiated.

In conclusion, the current extensive comparative PK and bronchodilator efficacy study in patients with COPD demonstrated a lower exposure but similar bronchodilator efficacy of once-daily tiotropium solution 5 µg compared with tiotropium powder 18 µg and supports the use of both approved tiotropium formulations for the maintenance treatment of patients with COPD.

## Declaration of Conflicting Interests

J.M. Hohlfeld is a paid consultant to Boehringer Ingelheim and has served on the Boehringer Ingelheim Advisory Board for Early Clinical Development. E. Derom is a paid consultant to Boehringer Ingelheim. J.A. van Noord is involved in carrying out contract research for Boehringer Ingelheim. A. Sharma, P.J.G. Cornelissen, L. Towse, V. Peterkin, and B. Disse are employees of Boehringer Ingelheim.

## Funding

Boehringer Ingelheim Pharma GmbH & Co KG and Pfizer Inc.

## References

[1] Celli BR, MacNee W (2004). Standards for the diagnosis and treatment of patients with COPD: a summary of the ATS/ERS position paper. Eur Respir J.

[2] From the Global Strategy for the Diagnosis (2011). Management and Prevention of COPD, Global Initiative for Chronic Obstructive Lung Disease (GOLD). http://www.goldcopd.org/.

[3] Bateman E, Singh D, Smith D (2010). Efficacy and safety of tiotropium Respimat SMI in COPD in two 1-year randomized studies. Int J Chron Obstruct Pulmon Dis.

[4] Bateman ED, Tashkin D, Siafakas N (2010). A one-year trial of tiotropium Respimat plus usual therapy in COPD patients. Respir Med.

[5] Maltais F, Hamilton A, Marciniuk D (2005). Improvements in symptom-limited exercise performance over 8 h with once-daily tiotropium in patients with COPD. Chest.

[6] Niewoehner DE, Rice K, Cote C (2005). Prevention of exacerbations of chronic obstructive pulmonary disease with tiotropium, a once-daily inhaled anticholinergic bronchodilator: a randomized trial. Ann Intern Med.

[7] Tashkin DP, Celli B, Senn S (2008). A 4-year trial of tiotropium in chronic obstructive pulmonary disease. N Engl J Med.

[8] van Noord JA, Cornelissen PJ, Aumann JL, Platz J, Mueller A, Fogarty C (2009). The efficacy of tiotropium administered via Respimat soft mist inhaler or HandiHaler in COPD patients. Respir Med.

[9] Vincken W, van Noord JA, Greefhorst AP (2002). Improved health outcomes in patients with COPD during 1 yr's treatment with tiotropium. Eur Respir J.

[10] Voshaar T, Lapidus R, Maleki-Yazdi R (2008). A randomized study of tiotropium Respimat soft mist inhaler vs. ipratropium pMDI in COPD. Respir Med.

[11] Hodder R, Price D (2009). Patient preferences for inhaler devices in chronic obstructive pulmonary disease: experience with Respimat Soft Mist inhaler. Int J Chron Obstruct Pulmon Dis.

[12] Durham MC (2004). Tiotropium (Spiriva): a once-daily inhaled anticholinergic medication for chronic obstructive pulmonary disease. Proc (Bayl Univ Med Cent).

[13] van Noord JA, Smeets JJ, Custers FL, Korducki L, Cornelissen PJ (2002). Pharmacodynamic steady state of tiotropium in patients with chronic obstructive pulmonary disease. Eur Respir J.

[14] Quanjer PH, Tammeling GJ, Cotes JE, Pedersen OF, Peslin R, Yernault JC (1993). Lung volumes and forced ventilatory flows. Report working party standardization of lung function tests, European Community for Steel and Coal. Official Statement of the European Respiratory Society. Eur Respir J Suppl.

[15] American Thoracic Society (1995). Standardization of Spirometry, 1994 Update. American Thoracic Society. Am J Respir Crit Care Med.

[16] Morais JA, Lobato MR (2010). The new European Medicines Agency guideline on the investigation of bioequivalence. Basic Clin Pharmacol Toxicol.

[17] Price D, Sharma A, Cerasoli F (2009). Biochemical properties, pharmacokinetics and pharmacological response of tiotropium in chronic obstructive pulmonary disease patients. Expert Opin Drug Metab Toxicol.

[18] Ichinose M, Fujimoto T, Fukuchi Y (2010). Tiotropium 5 microg via Respimat and 18 microg via HandiHaler; efficacy and safety in Japanese COPD patients. Respir Med.

[19] SPIRIVA® Respimat®, summary of product characteristics (2012).

[20] Dong YH, Lin HH, Shau WY, Wu YC, Chang CH, Lai MS (2013). Comparative safety of inhaled medications in patients with chronic obstructive pulmonary disease: systematic review and mixed treatment comparison meta-analysis of randomised controlled trials. Thorax.

[21] Karner C, Chong J, Poole P (2012). Tiotropium versus placebo for chronic obstructive pulmonary disease. Cochrane Database Syst Rev.

[22] Singh S, Loke YK, Enright PL, Furberg CD (2011). Mortality associated with tiotropium mist inhaler in patients with chronic obstructive pulmonary disease: systematic review and meta-analysis of randomised controlled trials. BMJ.

